# Dynamics and Functions of Stress Granules and Processing Bodies in Plants

**DOI:** 10.3390/plants9091122

**Published:** 2020-08-30

**Authors:** Geng-Jen Jang, Jyan-Chyun Jang, Shu-Hsing Wu

**Affiliations:** 1 Institute of Plant and Microbial Biology, Academia Sinica, Taipei 11529, Taiwan; Geng-Jen.Jang@jic.ac.uk; 2 Department of Horticulture and Crop Science, Department of Molecular Genetics, Center for Applied Plant Sciences, Center for RNA Biology, Ohio State University, Columbus, OH 43210, USA; jang.40@osu.edu

**Keywords:** stress granules, processing bodies, mRNA decay, translation

## Abstract

RNA granules, such as stress granules and processing bodies, can balance the storage, degradation, and translation of mRNAs in diverse eukaryotic organisms. The sessile nature of plants demands highly versatile strategies to respond to environmental fluctuations. In this review, we discuss recent findings of the dynamics and functions of these RNA granules in plants undergoing developmental reprogramming or responding to environmental stresses. Special foci include the dynamic assembly, disassembly, and regulatory roles of these RNA granules in determining the fate of mRNAs.

## 1. Introduction

A healthy balance of mRNA destinies, including decay, translation, and sequestration, is crucial for plants to optimize growth and development and combat biotic/abiotic environmental stresses. A plethora of mechanisms are responsible for mRNAs destined for degradation, including de-adenylation of the polyA tail and mRNA decapping followed by 5′ to 3′ decay, 3′ to 5′ mRNA decay, and co-translational decay [1,2]. The translation control of mRNAs often involves the formation of mRNA–ribonucleoprotein (mRNP) complexes, namely ribosomes/polysomes, and membraneless RNA granules formed via liquid–liquid phase separation (LLPS). LLPS is often triggered by proteins with intrinsically disordered regions that induce the aggregation of complex proteins or protein–RNA networks, such as stress granules (SGs) or processing bodies (p-bodies) [3,4]. SGs function primarily to store mRNA–ribosome complexes to halt translation, whereas functions of p-body components have been implicated in both mRNA decay and translation repression [5].

The protein constituents of SGs and p-bodies have been previously reviewed [6,7]. Briefly, in plants, p-bodies contain many RNA-binding proteins (RBPs) and key factors for mRNAs decay, including decapping complexes, 5′ to 3′ exoribonuclease, de-adenylation factors, small-RNA-dependent slicer protein Argonaute1, and factors involved in nonsense-mediated mRNA decay [7]. SGs are composed of polyadenylated mRNAs, translation initiating factors, the 40S ribosome subunit, RNA-binding proteins, and regulators of gene silencing [6]. A recent study involving the affinity purification of proteins and metabolites co-purified with the GFP-tagged SG marker Rbp47b has significantly expanded the protein repertoire of plant SGs [8]. In addition to the conserved components for SG assembly and dynamics, this study also discovered important regulators and stress-related proteins associated with SGs, including a key cell-cycle regulator cyclin-dependent kinase A. Intriguingly, this study also identified SG-associated metabolites, nucleotides, amino acids, and phospholipids, implying that SGs can also sequester small molecules [8]. Given that the composition of both SGs and p-bodies has been reviewed recently [6], the current review focuses on the recent advancements of the dynamic formation and functions of these two types of RNA granules in plants.

## 2. Stress Granules

SGs are membraneless organelles composed of proteins and translationally repressed mRNAs [9,10]. SGs are conserved in eukaryotes such as yeast, plants, and mammals [11,12,13]. In general, SGs are assembled when organisms face stresses and are often disassembled during recovery from the stress condition, which is accompanied by the restoration of active translation. In plants, multiple stresses (heat, hypoxia, high salt, and darkness), oxidative phosphorylation inhibitors (arsenite, potassium cyanide, and myxothiazol), and methyl jasmonate can trigger the formation of SGs [14,15,16,17,18]. 

The formation and functions of SGs under heat and hypoxia stresses have been better characterized and are summarized below.

### 2.1. SG and Heat Stress

**Figure 1:**
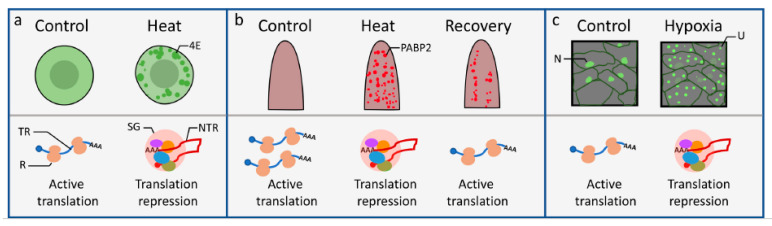
Stress granule dynamics and functions in translation repression in response to abiotic stresses. (**a**) Stress granules marked with eIF4E formed after 30 min of heat stress in tomato cells [17]. (**b**) Stress granules marked with RFP-PABP2 that accumulated after 1-h heat treatment and decreased after a 2-h recovery at 22 °C from heat stress in Arabidopsis roots [20]. (**c**) UBP1C-containing stress granules assembled in response to hypoxia stress in Arabidopsis leaves [16]. Note that the UBP1C-GFP subcellular localization pattern changed from the diffused distribution in the nucleus and cytoplasm to stress granules under hypoxia. For each subfigure, the upper panels illustrate the localizations of SGs within respective cells/tissues under mock (control), stress (heat, hypoxia), or recovery conditions. The lower panels are graphical representations of mRNA destinies correlating with the functions of SGs. 4E: eIF4E foci; R: ribosome; TR: translating mRNA; NTR: non-translating mRNA associated with the stress granule (SG); PABP2: RFP-PABP2 foci; N: nucleus; U: UBP1C-GFP focus.

When cells are exposed to high temperature stress, some proteins undergo misfolding and aggregation. In plant cells, heat shock proteins (HSPs), including the low-molecular-mass chaperones, HSP70 and HSP100, function together to interact with denatured proteins to prevent their aggregation and to disaggregate and refold the misfolded proteins [19]. On the other hand, SGs composed of mRNAs and translation initiation factor eIF4E were observed in tomato culture cells within 30 min of heat shock treatment [17] (Figure 1a). Under heat stress, translation is stalled, and pre-existing mRNAs are temporarily stored in SGs to reduce the protein influx that can be a burden to cells under heat stress. 

SGs often disassemble when recovering from stress conditions. For example, SGs were visualized by RFP-tagged SG marker polyA-binding protein 2 (PABP2) after 1-h heat treatment (37 °C), and the SG signals were reduced with a 2-h recovery from heat stress [20] (Figure 1b). In yeast, HSP104 (the homolog of Arabidopsis HSP101) and HSP70 are required to disassemble SGs after heat shock [21]. Similarly, the reduction of PABP2-associated SGs during the recovery phase was diminished in the *hsp101* knockout mutant, suggesting that HSP101 also functions to disassemble SGs during stress recovery in higher plants, thus allowing the translation to resume [20]. 

Under heat stress, the La-motif-related protein 1 (LARP1) functions as a heat-specific cofactor of the 5′ to 3′ exoribonuclease (XRN4) for a quick and co-translational degradation of mRNAs [22]. Of note, LARP1 primarily associates with SGs, and a small fraction of LARP1 co-localizes with XRN4 in p-bodies [22]. The possession of RNA-binding motifs and the multiple destinies of LARP1 implies a role in triaging mRNAs under heat stress.

Another type of cytoplasmic foci, the heat stress granule (HSG), was first identified in 1983 by electron microscopy as granular aggregations in heat-shocked (37 to 40°C) tomato suspension cells [11]. Small heat shock proteins (sHSPs) represent the key components of HSGs [11,23,24]. Biochemical data implied that these cytoplasmic HSGs were associated with specific mRNA populations and with ribosomes in their vicinity in heat-shocked tomato cells, especially during the recovery phase [24]. However, SGs and HSGs are two distinct structures, and mRNAs are predominantly associated with SGs [17]. 

### 2.2. SGs and Hypoxia Stress

Translation initiation is strongly reduced in Arabidopsis seedlings under hypoxia stress [25,26]. In mammals, T-cell intracellular antigen 1 (TIA-1) and TIA-1-related (TIAR) proteins are key components in SG assembly [27,28]. In plants, OLIGOURIDYLATE BINDING PROTEIN 1C (UBP1C) has a high sequence identity with TIA-1 and TIAR proteins and is essential for the growth and survival of Arabidopsis under hypoxia stress. UBP1C has 3 RNA recognition motifs that can bind and sequester mRNAs to SGs induced by oxygen/ATP deprivation (e.g., hypoxia) in Arabidopsis, a process coinciding with the global decline of protein synthesis [16] (Figure 1c). 

### 2.3. Regulation of SG Dynamics and Movement

SGs are highly dynamic, mobile structures. A recent study elegantly revealed the temperature threshold needed for SG formation [29]. In the absence of transpiration to reduce the plant body temperature, SGs were formed at a critical temperature of 34 °C. The size and number of SGs also varied in response to the intensity and duration of heat shock treatment. This study also showed that actin filament is responsible for the long-distance movement of SGs in Arabidopsis cells [29]. A fine balance between the polymerization and depolymerization of microtubules is also important for the assembly of SGs [14].

The degradation of RNAs under stress conditions may lead to the production of 2′,3′-cAMP, a conserved small molecule among organisms in different kingdoms [30,31,32]. The search for 2′,3′-cAMP-interacting protein partners identified polyadenylate-binding protein Rbp47b, one of the major components in SGs [17,27,33]. 2′,3′-cAMP alone can promote the formation of SGs, providing evidence for small-molecule-induced SG assembly [33].

## 3. P-Bodies

Mutations in most p-body components have led to postembryonic lethality in Arabidopsis [34], suggesting that core components of p-bodies are crucial for successful developmental transition during an early developmental program. The composition and inferred functions of p-bodies in development and stress responses have been comprehensively documented [7]. Below we focus on recent advances in the mechanistic characterization of p-body components in executing the functions of mRNA decapping/decay and translation repression and the biological relevance of such functions for development and responses to both biotic and abiotic stresses.

### 3.1. P-Body-Mediated Translation Repression and Developmental Regulation

One internal factor regulating early seedling development is the gaseous phytohormone ethylene [35]. One branch of the ethylene-mediated signaling pathway involves the p-body-mediated translation repression of two key negative regulators of the ethylene signaling pathway: ethylene insensitive 3 (EIN3)-binding F-box1 (EBF1) and EBF2 [36]. EIN2 functions as a bridge to associate EBF1/2 mRNAs with p-bodies via dual interaction capacities with the polyU structure in the 3′ untranslated regions (UTRs) of EBF1/EBF2 mRNAs and p-body components XRN4/EIN3 and polyA-binding proteins (PABs) [36]. Consistent with the translation repression role of p-bodies in EBF1/EBF2, Arabidopsis mutants defective in multiple p-body components showed ethylene-insensitive phenotypes [36]. 

**Figure 2:**
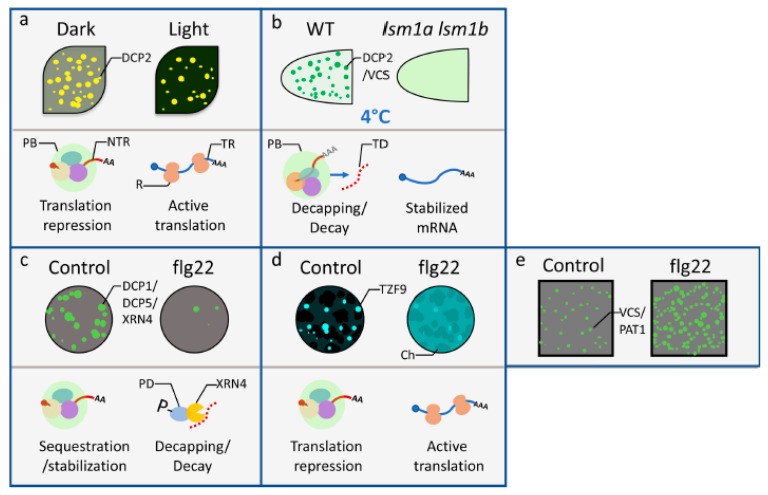
P-body dynamics and functions in mRNA decay and translation repression in response to abiotic or biotic environmental cues. (**a**) P-bodies and associated global translation repression in Arabidopsis cotyledons decreased during transition from dark to 4-h light treatment [37]. (**b**) Low temperature induced formation of p-bodies and specific mRNA decay in Arabidopsis root tips was dependent on LSM1A and LSM1B [40]. (**c**) Yu et al. (2019) demonstrated that the treatment of flg22 triggered the disassembly of DCP1-associated p-bodies and differential mRNA decay in Arabidopsis protoplasts [42]. (**d**) The treatment of flg22 led to the decrease of TZF9-containing granules and the release of translation repression in Arabidopsis protoplasts [43]. The spherical dark-shade clusters in the background of the upper panel of (d) are chloroplasts (Ch). Note that the TZF9 fluorescence signal changed from distinct granules to a diffusive cytoplasmic pattern. (**e**) In contrast to (c), the treatment of flg22 induced the formation of PAT1-associated p-bodies in Arabidopsis root epidermal cells [44]. For each subfigure, the upper panel illustrates the localization of SGs within the respective cells/tissues under mock (control) or treatment (light, flg22) conditions. The lower panels are graphical representations of mRNA destinies correlating with the functions of p-bodies. DCP2: DCP2-YFP focus; DCP2/VCS: GFP-DCP2 or GFP-VCS focus; DCP1/DCP5/XRN4: DCP1-GFP, DCP5-GFP, or XRN4-GFP focus; TZF9: TZF9-GFP focus; VCS/PAT1: VCS-GFP or PAT1-GFP focus; PB: p-body; NTR: non-translating mRNA associated with PB; R: ribosome; TR: translating mRNA; TD: target of decapping machinery; P: phosphate; PD: phosphorylated DCP1.

More recently, p-bodies were found to play a key role in controlling the timely developmental switch from skotomorphogenesis to photomorphogenesis [37]. Comparative transcriptome and translatome analyses of seedlings of the wild type and an Arabidopsis mutant defective in *Decapping 5*, *dcp5-1*, revealed that p-bodies could sequester and defer the translation of thousands of mRNAs. The temporary storage of mRNAs prohibits the premature translation of proteins for apical hook opening or biosynthetic enzymes producing excessive amounts of harmful pigments, such as protochlorophyllide. However, the exposure of etiolated seedlings to light triggers a reduced accumulation of p-bodies to release the translationally stalled mRNAs for the active translation of mRNAs encoding proteins needed for photomorphogenesis [37] (Figure 2a).

### 3.2. P-Body-Mediated Decapping/Decay for Abiotic Responses

Both SGs and p-bodies can sequester mRNAs and stall their translation. However, p-bodies (but not SGs) are equipped with RNA-degradation machineries. Therefore, in addition to the translation repression roles, p-bodies have been indirectly implicated in the decapping and degradation of mRNAs such as *EXPL1*, *SEN1*, multiple transcription factor mRNAs [38], and those encoding seed storage proteins [39]. In addition, despite p-bodies primarily exerting translation repression in etiolated seedlings, increased abundance was also observed for hundreds of mRNAs in etiolated *dcp5-1* seedlings [37]. These results imply that DCP5 may have an additional function in facilitating mRNA decay. Whether the dual roles of DCP5 in translation repression and mRNA decay are executed in p-bodies, developmentally regulated, and/or environmental condition dependent requires further study.

The decapping/decay capacities of p-body components have contributed to multiple abiotic responses in plants. For example, Arabidopsis carrying mutations in p-body-associated decapping activators and de-adenylation protein complexes led to defects in abiotic stress responses [40,41]. In addition, mutations in the decapping activator SM-like protein LSM1-7 complex resulted in reduced p-bodies under low temperature stress and increased freezing tolerance [40] (Figure 2b). Among the candidate decapping/decay targets are transcripts of cold-induced *9-cis-epoxycarotenoid dioxygenase* 3 (*NCED3*) and *NCED5*, two key genes for abscisic acid (ABA) biosynthesis. In the *lsm1a lsm1b* double mutant, stabilized *NCED3* and *NCED5* mRNAs led to increased ABA levels and cold tolerance [40]. This implied that, in wild-type plants, the LSM complex functions to tune down the expression of *NCED3* and *NCED5* at the post-transcriptional level to ensure an optimal ABA level for achieving a balance between growth and cold tolerance.

P-bodies were also induced to form under osmotic stresses. Under osmotic stresses, another class of p-body-associated decapping activator, VARICOSE (VCS), was found to be phosphorylated by SNF1-related protein kinase 2 (SnRK2) and responsible for the decapping and decay of hundreds of mRNAs, a process that is necessary to maintain plant growth and development under osmotic stresses [45]. These SnRK2-VCS pathway functions are independent of ABA, suggesting a timely regulatory role of p-bodies in modulating the transcriptomic shift before ABA accumulation. 

The number of p-bodies increases in Arabidopsis under salt stress. The assembly of p-bodies under salt stress depends on the presence of a DCP1-interacting, p-body-localized BEACH-domain-containing protein known as SPIRRIG (SPI) [46]. Without SPI, Arabidopsis plants are hypersensitive to salt stress. Transcriptomic comparisons between the wild type and *spi* mutant indicated that SPI regulates the abundance of some salt-stress-regulated mRNAs, possibly by targeting and stabilizing them in p-bodies [46].

### 3.3. P-Body Assembly, mRNA Decay, and Translation Repression in Plant Immunity

Arabidopsis carrying mutations in the de-adenylation protein complex associated with p-bodies have defects in abiotic stress responses [41] and biotic stress responses [47]. In addition, a recent study indicated the positive roles of the p-body components DCP1 and DCP2 in plant immunity [42]. The elicitation of plant pattern-triggered immunity (PTI) by microbe-associated molecular patterns (MAMPs) led to a quick and transient disassembly of p-bodies and mitogen-activated protein kinase 3 (MPK3)/MPK6-dependent phosphorylation of DCP1 on Ser237. Phosphorylated DCP1 had higher affinity with XRN4, a 5′ to 3′ exonuclease, to degrade the negative regulators of plant immunity [42] (Figure 2c). The quick removal of these negative regulators by decapping and degrading corresponding mRNAs granted the plants timely responses to pathogen invasion. 

In response to flagellin (flg22) treatment, MPK3/MPK6 phosphorylated another p-body component, tandem zinc finger protein 9 (TZF9), and led to a decrease of TZF9-associated p-bodies in Arabidopsis protoplasts [43] (Figure 2d). A comparison of the transcriptome and translatome between the wild type and *tzf9* mutant revealed that TZF9 can regulate flg22-dependent gene expression at the translation level [43]. Together with data from Yu et al. [42], the treatment of flg22 induced the disassembly of p-bodies in both cases. However, in this process, different mRNAs originally associated with p-bodies appeared to have different fates. Those destined to be degraded include the mRNAs encoding the negative regulators of the plant immune responses elicited by flg22 [42]. On the other hand, those resuming active translation may encode the positive regulators of early immune responses. The flg22-triggered dynamic disassembly of the p-bodies triages the fates of sequestered mRNAs of different functionalities, which together contribute to the robustness of plant early immune responses.

In addition to positive regulators, such as MPK3/MPK6 in plant immunity, the flg22 treatment also activated a negative regulator of plant immunity, MPK4, to phosphorylate a decapping enhancer, the Arabidopsis homolog of yeast PAT1 (PAT1) [44]. However, in contrast to the flg22-induced decrease of p-bodies in protoplasts, Arabidopsis roots showed increased p-bodies with flg22 treatment (Figure 2e). Whether this finding represented kinetic differences between leaf protoplasts and root tips remains to be carefully examined. Additionally, whether PAT1 and DCP1/XRN4 target different sets of plant immunity mRNAs for decay is unknown, as is how the fine balance of mRNA decay and translational control mediated by p-bodies could together optimize plant immunity.

**Figure 3:**
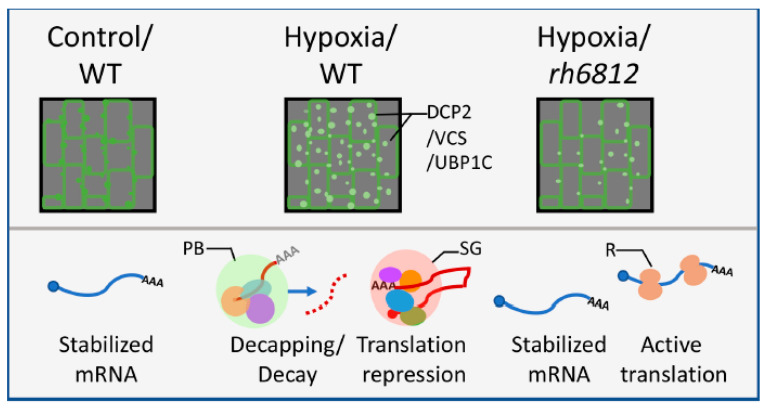
The dynamics and functions of p-bodies and SGs are regulated by RNA helicases. The numbers of p-bodies (marked by DCP2 or VCS) and SGs (marked by UBP1C) increased in response to coverslip-induced hypoxia in Arabidopsis root epidermal cells, a process requiring specific RNA helicases (RHs) [48]. Note the reduction of cytoplasmic granules in *rh6812* triple mutant under coverslip-induced hypoxia. The induction of these RNA granules was correlated with selective mRNA decay and translation repression. For each subfigure, the upper panel illustrates the localization of SGs within wild-type cells/tissues under mock (control) or hypoxia conditions and the *rh6812* mutant under hypoxia condition. The lower panels are graphical representations of mRNA destinies in the corresponding cells/tissues. WT: wild-type Arabidopsis; DCP2/VCS/UBP1C: DCP2-GFP, VCS-GFP, or UBP1C-GFP focus. PB: p-body; SG: stress granule; R: ribosome.

A recent report integrating sophisticated transcriptome and translatome analyses showed that an Arabidopsis mutant defective in DEAD-box RNA helicase 6 (RH6), RH8, and RH12 showed preferential accumulation and translation of stress- and defense-related mRNAs [48]. RHs are part of the decapping-dependent 5′ to 3′ mRNA decay machinery and were found to co-localize with both p-body and SG markers [48]. Under normal conditions, these RHs function to keep the stress- and immunity-related mRNAs at basal levels so that the plant cells can allocate resources to mRNAs for growth and development (Figure 3). Consistent with this notion, the Arabidopsis *rh6812* triple mutant exhibited constitutive innate immunity at the expense of growth and development [48]. Of note, although the assemblies of both p-bodies and SGs were defective in the Arabidopsis *rh6812* triple mutant [48] (Figure 3), the decay of VCS-dependent mRNAs was largely unaffected [48], so mRNA decay may occur co-translationally or in p-bodies of sizes undetectable by current microscopy technology. 

A viral infection often triggers small-RNA-mediated gene silencing in plants. Argonaute1 is a key protein in gene silencing and is also a component of p-bodies. Evidence for the involvement of p-bodies in the plant–viral interaction has been comprehensively described in a previous review [49].

## 4. Future Perspectives

Both SGs and p-bodies represent phase-separated protein–RNA condensates. Defects in the formation/assembly of SGs and p-bodies have been observed in mutants defective in many RBPs. Whether these RBPs are directly involved in the assembly of the RNA granules or the defects in assembly are caused indirectly by translation inhibition in RBP mutants remains to be clarified. A recent study may provide a hint to support the role of RHs in SG/p-body assembly. This study reported that the evolutionarily conserved RH homologs RNA-dependent DEAD-box ATPases (DDXs) could promote the formation of phase-separated organelles in both in vitro and in vivo conditions. These DDXs could also control the RNA flux into and out of the phase-separated organelles [50]. Whether plant RHs share the same properties in phase separation and RNA partition needs to be studied in the future. 

Another unresolved issue is whether p-bodies are sites for mRNA decay. By using RNA fluorescence in situ hybridization, β-actin mRNAs levels were found to be increased within p-bodies in Dcp2-knockdown mammalian cells [51], suggesting that β-actin mRNAs were degraded in the p-bodies. However, increasing evidence has argued otherwise. For example, p-body-associated mRNAs derived from a reporter construct were mostly intact in mammalian cells under normal or short-term stress (1-h arsenite treatment) conditions [52]. A similar observation was also made for endogenous mRNA populations associated with p-bodies via marker-assisted sorting and purification from mammalian cells under a non-stressed condition [53]. The latter study pointed to a more restricted role for p-bodies in mRNA storage and translation repression. Whether plant p-bodies function primarily in mRNA storage, as in mammals, and/or are directly involved in the decay of specialized mRNAs under certain developmental or stress conditions requires additional study.

Despite the potential importance of SGs and p-bodies in mRNA triage, many key mechanistic aspects remain to be understood. Although the composition of SGs and p-bodies has been heavily investigated, their complete proteomes under dynamic internal and external cues remain to be unveiled. To achieve this goal, high-throughput and high-resolution approaches such as SG/p-body purification [54] and protein proximity labeling [55,56] could be adopted. Furthermore, RBPs play a central role in targeting mRNAs; however, very little is known about the mechanisms of RBP–RNA interactions and the resulting transcriptome sequestered to SGs and p-bodies. Recent development of high-throughput UV crosslinking immunoprecipitation coupled with RNA-seq (CLIP-seq) could help decipher the RNA elements targeted by RBPs [57]. 

In addition, we need to determine how specific functional groups of mRNAs are recruited to SGs/p-bodies and the underlying signaling mechanisms regulating the dynamic formation of the RNA granules in plant cells. This research will require in-depth characterization of the protein candidates contributing to liquid–liquid phase separation as well as mRNAs carrying nucleotide modifications and/or unique sequence signatures (*cis*-RNA element, RNA motif structure, or both). For example, a recent study in mammalian cells indicated that mRNAs carrying N^6^-methyladenosine (m^6^A) could drive the phase separation potential with the help of the m^6^A-binding proteins YTHDF1, YTHDF2, and YTHDF3 [58]. Of note, an Arabidopsis m^6^A reader, Evolutionarily Conserved C-terminal Region 2 (ECT2), also carries an aggregation-prone YPQ-rich motif and can relocate from the cytoplasm to SGs under heat stress [59]. 

Besides dynamic assembly/disassembly, membraneless RNA granules are highly mobile in diverse organisms. In mammals, the mobility of both p-bodies and SGs relies on microtubules [60,61]. In plants, both microtubules and actin filament are involved in the formation and transport of SGs [29]. Actin filament is required for the long-distance transport and formation of large SGs under high temperature [29]. The movement of p-bodies is also connected to the actions of actin filament [62] and myosins via interaction with DCP1 [63]. Additional efforts are needed to delineate mechanisms for the delivery of mRNAs/proteins to RNA granules and the movement of RNA granules within plant cells.
